# An exploratory study of addressing bias for child abuse teams: the role of narrative medicine

**DOI:** 10.3389/fped.2025.1710240

**Published:** 2026-01-14

**Authors:** Jocelyn Brown, Talea Cornelius, Gabriella Farland, Philip Gialopsos, Rita Charon

**Affiliations:** 1 Department of Pediatrics, Vagelos College of Physicians & Surgeons Columbia University Irving Medical Center, New York, NY, United States; 2 Center for Behavioral Cardiovascular Health, Columbia University Irving Medical Center, New York, NY, United States; 3 eMAX Health, New York, NY, United States; 4 Lenox Hill Hospital, Department of Medicine, New York, NY, United States

**Keywords:** child abuse teams, exploratory study, health humanities, implicit bias, narrative medicine

## Abstract

**Introduction:**

Art and humanities-based approaches have been incorporated in Diversity, Equity and Inclusion (DEI) training and anti-bias curriculum to address structural racism and personal biases via reflection. Research has shown that the use of visual art or texts via narrative medicine workshops results in improved communication with patients and colleagues and increased commitment to interrupting bias.

**Methods:**

Using members of a hospital-based Child Abuse Bias Mitigating Task Force, this study tests the hypothesis that narrative medicine workshops provide a space where conversations of race and bias in the context of child abuse evaluations can take place.

**Results:**

Workshops participants noted the unique group experience that, through sharing and communal support, helped build compassion, function more effectively as a team, and even find confidence in their own voice. Intertwined with the ability to connect with and support each other as a team was the common thread of understanding differences in perspectives and personal histories. Most participants agree that the workshops increased their ease in having conversations about privilege and bias in clinical assessment.

**Conclusion:**

We conclude that the use of art and creativity allows for personal and structural insights on racism and social advocacy with significant promise for reducing bias in child abuse evaluations.

## Introduction

There is ample evidence that both structural racism and health care providers' own biases affect medical decisions and treatment options in clinical care (1, 2). The impact of these factors on physician evaluation of families for suspicion of child abuse and neglect is an urgent public health issue. Racial disparities and bias and variability in diagnosing abuse have been identified as research priorities by a prospective, multi-center child maltreatment research network (3). Clinicians often rely on their perception of risk and on their “gut-feeling,” resulting in overevaluation, overreporting, and more removals of children from Black and brown families than from their White counterparts (4, 5). Though some of these perceptions are influenced by explicit biases, other biases are more implicit and insidious.

Investigation by child protective services is not equal across race. Using the National Child Abuse and Neglect Data System (NCANDS), Kim showed that Black families had the highest rate of investigation by child protective services and Asian/Pacific islander the lowest (6). Racial disparities have been understood along two different and competing models. According to the model of higher risk factors (62, 63), Black children have a higher concentration of risk factors such as economic insecurity that put them at risk of maltreatment. According to the model of racial bias, interpersonal and structural racism contribute to Black children being reported and investigated at a higher rate than other children (7). Overall, research supports the relationship between poverty and child maltreatment, but it does not explain the variance seen in these cases.

Research to-date shows that mandated reporters are influenced by their perceptions of poor and minorities families (4) and that structural racism contributes to disparate risk of maltreatment, resulting in disparities in child protective services investigations and disproportionalities in the child welfare system (7). Evaluation, diagnostic decision-making, and reporting of child abuse vary along the lines of social class and race (3). Pediatricians are more likely to suspect abuse in Black and Latinx families (8, 9) and to obtain radiologic tests such as skeletal survey in Black children (10). The relationship of bias and child maltreatment is complex showing in some studies bias towards race but not class (11) or class but not race (12). Race and class can be confounded and race can be a proxy for class. Hymel et al. (11) showed that children of color admitted to an intensive care unit for head trauma were more likely to be evaluated and reported for abuse despite a lower risk of abusive head trauma based on a validated clinical prediction rule tool (13). However this finding was only observed in 2 out of the 18 sites, raising the question of possible implicit bias among the providers. Zamalin et al. show that for cases with low likelihood of child physical abuse, clinicians disagree with Child Abuse Pediatricians in 38% of the cases and were more likely to suspect abuse of families with low socioeconomic status (SES) and prior history with child protective services (12). It is interesting to note that, in both studies, suspected bias occurs primarily with cases with low likelihood of abuse. We do not know whether this bias is the result of a perception of a higher risk based on the family characteristics (4) or a gray case in which there was uncertainty in formulating a medical diagnosis (14).

Implicit bias is difficult to measure. It is also important to recognize that making the decision to report and diagnose abuse cases is stressful and can be emotionanly charged. There is also evidence that the diagnosis of abuse is overlooked and unaccounted for in White families. In Jenny's seminal article (15), abusive head trauma was likely to be unrecognized in very young White children (v. minority children) and children from intact families. The societal costs of overdiagnosing or underdiagnosing abuse are high. Families of color unfairly experience the additional emotional stress of investigation. Underrepresented minority children receive unnecessary exposure to radiation, while White children are likely to be re-abused and re-injured when child abuse is missed (6, 10, 15, 16). In sum, research shows that clinicians have different threshold levels to suspect, evaluate, and report child abuse leading to bias and disparities in the system. Addressing these biases at the front door are key to reducing downstream disparities in the child welfare system (17). Individual strategies, such as deliberate reflection, are strategies used to address implicit bias (18).

Many institutions have implemented Diversity, Equity, and Inclusion (DEI) training for bias mitigation, but these trainings often cannot fully address the difficult and emotionally fraught experience of making a diagnosis of abuse. Beyond the already-difficult conversations about race and racism, for which many medical professionals are unprepared (19), facing the reality of a child having been abused with the pressure of being a mandated reporter can be overwhelming (20). New approaches that appreciate and incorporate the role of reflection and processing in addressing bias in child maltreatment cases are needed. Organizations such as the Association of American Medical Colleges (21) and the National Academy of Sciences, Engineering and Medicine (22) have recognized that arts and humanities are an integral part of medical education. Furthermore, recent literature shows that the arts and the humanities provide a framework to address advocacy and social justice (23). Art and humanities-based approaches have been used to address structural racism and uncover personal biases via reflection (24) allowing for difficult conversations to take place. Towards that end, Balhara and colleagues developed a health humanities-based longitudinal curriculum for emergency medicine residents and faculty which resulted in improved communication with patients, colleagues and increased commitment to interrupting bias and systematic racism (25).

## Team-building with narrative medicine

Narrative medicine introduces humanities methods into clinical training and practice to expand clinicians’ insight and collaboration. Defined in 2001, narrative medicine proposes that “the abilities to acknowledge, absorb, interpret, and act on the stories and plights of others” are necessary capacities for clinicians and others in human services (26). Narrative medicine training increases clinicians' capacity for attention to others' perspectives by reading creative texts together and writing responses to understand one another's concerns. Doing narrative work in groups deepens affiliation among members of the group (27). Systematic reviews since 2017 have documented outcomes of this pedagogy (28, 29).

We used these admittedly unconventional methods from humanities and creative arts to encourage clinicians to listen to one another's perspectives and to build trust and open personal dialogue among members of their multidisciplinary child abuse team. As documented in the qualitative analysis in this study, participants gradually heard one another's perspectives instead of retaining their own explicit or implicit biases. We propose that these outcomes will improve team members' capacity to deliver balanced and informed assessments in clinical decision-making.

Although these methods are only now being introduced into child abuse settings, related fields of clinical oncology practice and medical education have adopted narrative methods to improve individual and group insight, perspective-taking, and team cohesion. These models support our adoption of narrative methods in child abuse training and evaluation to strengthen team cohesion and mutual shared perspectives, thereby minimizing decision-making on the basis of individuals’ implicit biases.

Paul et al. (30) systematically reviewed narrative interventions in clinical oncology practice. The following studies achieved high ratings on the McGill Mixed Methods Appraisal Tool (MMAT) and report settings and findings salient to our work in child abuse team-building. Two studies by Saint-Louis reported improvements in shared perspectives among inter-professional teams in out-patient and in-patient settings in the US (31, 32). Sands et al. (33) reported on increased teamwork, increased empathy, and decreased burnout among inter-professional pediatric oncology team members in a mixed-method cohort study. Richardson et al. (34) reported increased solidarity among medical oncology fellows in a mixed-method cohort study.

Milota and colleagues performed a systematic review of narrative-based methods in medical education settings (35). They propose that “a narrative medicine classroom intervention entails encountering and/or analyzing an art form or narrative, reflecting upon this encounter, and sharing one's discoveries with others in carefully monitored and supportive environment” (p. 3). Their overall assessment of the educational criteria achieved of studies reviewed includes modification of attitudes, perceptions, knowledge, or skills, based on the Best Evidence in Medical Education (BEME) criteria for educational achievement in medical education. Examples of such modifications are increased identification with their peers, better awareness of cultural diversity and enhanced understanding of and capability in communication.

Outcomes studies of narrative medicine training in other healthcare settings reveal increases in self-awareness, non-judgmental listening to accounts of others, and team cohesion (36). Educational courses using narrative medicine techniques have been shown to strengthen students' reflection, patient-centeredness, and perspective-taking (37, 38). Narrative medicine trainings in eating-disorder settings among faculty and patients resulted in allyship with patients, a sense of recognition, embrace of uncertainty, and fostering agency (39).

Although few DEI initiatives have utilized narrative medicine as a framework specifically to inform DEI training, narrative medicine workshops show significant promise for addressing existing gaps by incorporating themes of race, social justice, and structural violence (40). Narrative methods have been adopted for implicit bias training and anti-racism awareness among clinicians and trainees (25). After an intensive 3-day anti-racism workshop using the methods and principles of narrative medicine, Charon et al. (41) demonstrated an impact on bias reduction of the aesthetic and creative dimensions of this approach. Others have documented similar consequences for students in reflecting on and processing of racial dimensions (42). The concept of “abolition medicine” has increasingly been adopted to express the fundamental anti-racist nature of narrative medicine methods in health care (64). The use of explicit visual art such as “Emergency Room” by Robert Colescott can generate conversations of injustice and racism in medicine (43).

Such outcomes as improvement in team trust, increased willingness to disclose one's impressions of a clinical case, and being prepared to discuss one's assessment of a case with other members of a team reduce the likelihood of biased decision-making and enable more nuanced avenues toward balanced decisions.

## The present study

The primary aims of this project were (1) to test the feasibility of implementing a 6-session narrative medicine workshop program to strengthen team cohesion across a range of disciplines working with child abuse in a medical setting and (2) to collect qualitative data testing the hypothesis that narrative medicine workshops provided a safe space where conversations about racial, cultural and social bias in the context of child abuse evaluations could take place. The workshops were planned in accordance with viewpoints of abolitionist medicine, whose “essential work … is to interrogate the upstream structures that enable downstream violence …, reimagining the work of medicine altogether as an anti-racist practice” (64). Protection of the health of children in the United States occurs in the historical context that countenanced the racism introduced in slavery and has been maintained since the abolition of slavery in countless forms of social life (44, 45). This project intentionally engaged multi-racial members of inter-professional health disciplines in hard conversations about race, parenting, familial loss, Black culture, and justice. By establishing safety through the early adoption of group norms, the group grew to articulate complex recognitions of the social implications of their actions in child abuse medicine and to challenge their own impressions about race with the contrasting viewpoints of others in the group. Over the longitudinal group process spanning 3 months, participants joined in examining the social and cultural contexts of the families they serve, gradually strengthening their own recognition of the power hierarchies within which they and their patients work and live.

## Methods

### Participants

In 2020 the Columbia University Irving Medical Center (CUIMC) Department of Pediatrics established a Child Abuse Bias Mitigation Task Force (CAB-MTF) during a period of nationwide racial unrest following the death of George Floyd and the rise of the Black Lives Matter movement in the U.S. The initiative stemmed from a growing awareness of racial disparities in child abuse reporting within hospital settings and the necessity of reducing bias and promoting equity in the identification and management of child abuse. Following an award of an advocacy grant by the Columbia Children's Health Innovation Nucleation Fund, the initiative was announced at a Department of Pediatrics faculty meeting, where clinicians volunteered to join the committee. The group, which includes pediatricians and social workers from outpatient, ICU, hospitalist, and emergency settings, comprised members actively involved in education, diversity and equity efforts, narrative medicine training, or the child protection team. During the first year, members were trained on child abuse evaluations during monthly meetings and were invited to bring cases for discussion at bi-weekly meetings if they had any concern about bias. After this first year of training, the 24 members of the CUIMC CAB-MTF were invited to participate in six 1-hour narrative workshops to continue their training in equitable and bias-limited child abuse decision-making.

### Procedure

The planning group, composed of the Director of CAB-MTF, a group facilitator trained in narrative medicine, and two research associates, identified the broad topics to be visited in the course: parenthood, Black culture, multi-racial relationships, familial loss, and white impressions of Black culture. Guided by the facilitator, the planning team chose texts based on their salience to the topics, the goals of the workshop, and the text's suitability for shared examination. The participants, the facilitator, and a research staff note-taker (who did not participate in the conversation) joined via Zoom for 1-hour sessions held six times during the study period at 7- or 14-day intervals. Group membership fluctuated depending on conflicting individual members' clinical duties. The facilitator presented a written text or a visual image chosen by the research team. Participants discussed the text/image, voicing their own impressions and interpretations, with the facilitator providing background information on the artist or writer of the text or image. Part-way into the hour, the facilitator provided an expansive writing prompt and invited participants to free-write in response to the prompt for about 5 min. At the end of the writing period, participants were encouraged to read aloud or describe what they had written [A syllabus of written/visual materials and writing prompts is included in supplemental material (Appendix A)]. Participants received a $50 Gift Card for participation; participants were able to select where this Gift Card applied. Compensation was dependent on survey completion (baseline, post-workshop) and attending at least one Zoom session.

Participants were invited to complete three surveys via Qualtrics. Links were sent by email, and three reminders were sent if surveys were not completed. The baseline survey queried demographic data and selected quantitative measures about racial bias (not included in the present manuscript). The post-workshop survey again queried quantitative measures. A third post-workshop survey included questions about the acceptability, feasibility, and appropriateness of this workshop for reducing bias in medicine and five free-text questions designed to elicit participants' reflections on the impact of the workshops. Only participants who attended at least one workshop were invited to complete follow-up surveys.

### Measures

#### Recruitment

Recruitment rate was computed as the proportion of participants who enrolled in the study out of those invited.

#### Attendance

Number of sessions attended was tabulated for each participant.

#### Compliance

Compliance was computed as the number of participants who completed each survey assessment.

#### Acceptability

Acceptability was assessed using the 4-item Acceptability of Intervention Measure [AIM; e.g., “I like the Narrative Medicine Workshop(s)”] (46). Response options ranged from 1, *completely disagree*, to 5, *completely agree*, with higher mean scores indicating greater acceptability. A cutoff ≥4 was pre-specified as indicating agreement that the narrative medicine workshops were acceptable.

#### Feasibility

Feasibility was assessed using the 4-item Feasibility of Intervention Measure (FIM; e.g., “The Narrative Medicine Workshop seems implementable”) (46). Response options ranged from 1, *completely disagree*, to 5, *completely agree*, with higher mean scores indicating greater feasibility. A cutoff ≥4 was pre-specified as indicating agreement that the narrative medicine workshops were feasible.

#### Appropriateness

Appropriateness was assessed using the 4-item Intervention Appropriateness Measure [IAM; e.g., “The Narrative Medicine Workshop(s) seems like a good match for reducing racial bias in medicine”] (46). Response options ranged from 1, *completely disagree*, to 5, *completely agree*, with higher mean scores indicating greater appropriateness. A cutoff ≥4 was pre-specified as indicating agreement that the narrative medicine workshops were appropriate.

#### Qualitative

Open-ended questions have been utilized to collect qualitative feedback for educational workshops in the Narrative Medicine Master of Science Program at Columbia University. The first four general questions listed here are appropriate to query our participants in this child abuse project. We added a fifth question specifically targeting this training in our child abuse setting. Questions were:

Please tell me about a moment that stood out in one of the sessions of the narrative medicine workshop.
What do you think changed, if anything as a result of having these sessions?
What did you learn about your team members in the process?
What did you learn about yourself in the process?
Do you think it is easier to have conversations of privilege and bias in your clinical assessment as a result of these sessions?

### Data analysis strategy

For attendance, we computed the mean and standard deviation (SD) number of sessions attended and the proportion of participants attending at least one session. Mean (SD) for participant report of acceptability, feasibility, and appropriateness was computed, as was the proportion classified as agreeing with each of these metrics. Given the small sample size, it is not possible to draw statistical conclusions about any potential differences in ratings according to session attendance, and readers are cautioned against doing so; however, these metrics are reported separately for those attending one session only vs. more than one session for completeness.

Open-ended survey responses were compiled into a single document transcript. Following a modified grounded theory approach (47–49), two researchers (TC, GF) reviewed all participant comments to identify key themes. The researchers then compared notes and developed themes through discussion. The structure of these themes resulted in two over-arching themes with four sub-themes each (one for each open-ended question, items 1–4) and one theme indicating agreement/disagreement with question 5 (see Table 3).

After finalizing the codebook and theme definitions, all comments were coded independently by the two researchers. Codes were applied in chunks with no specified length; rather, a “chunk” was defined to be a question response of any length expressing a complete thought. It was possible for one written comment to be double-coded (i.e., to contain more than one theme). After coding was complete, the two researchers met to discuss codes and reconcile coding discrepancies. The raters had fair agreement (Cohen's Kappa = 0.3).

## Results

**Figure 1:**
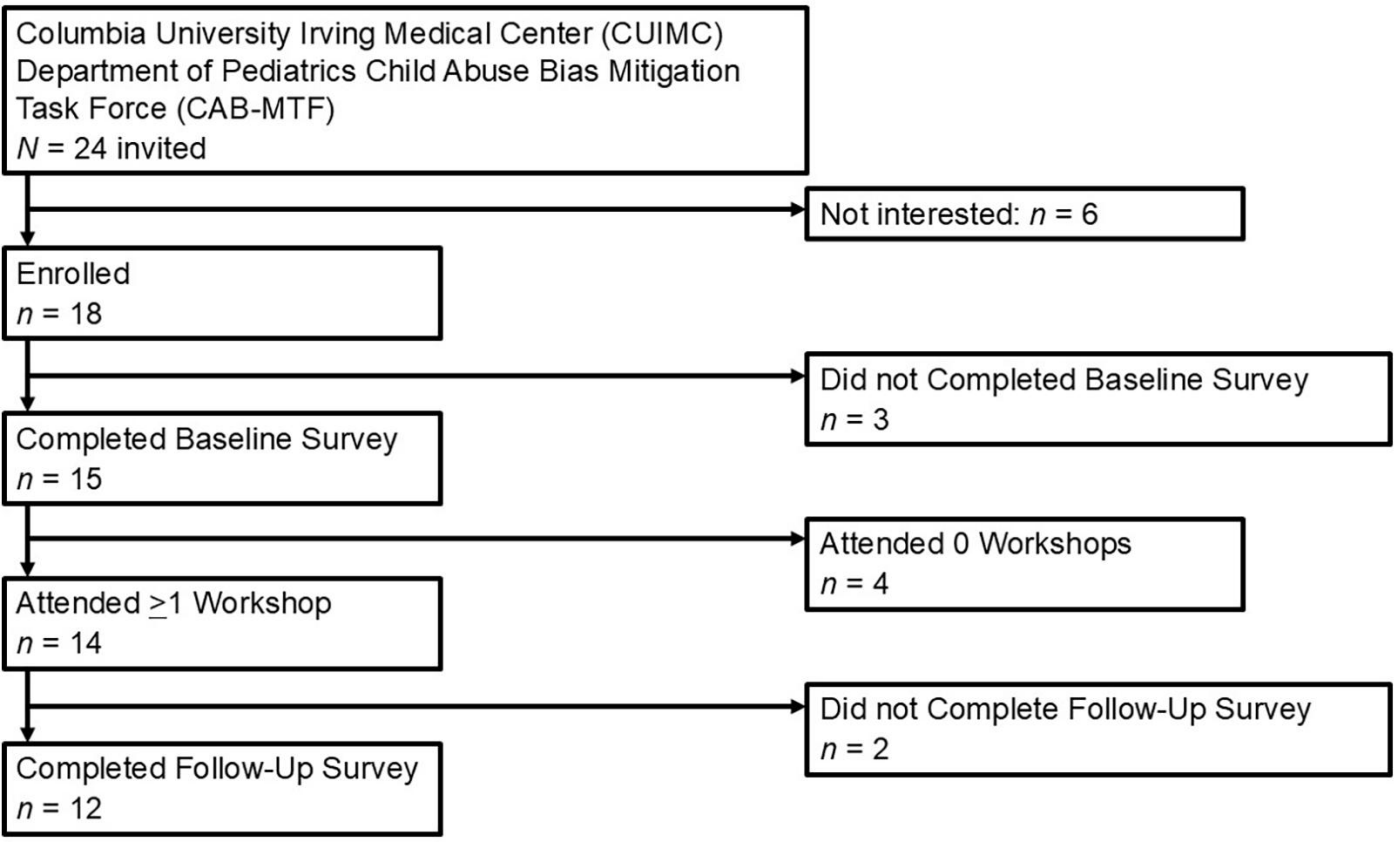
Participant flow.

Of the 24 CAB-MTF members invited to participate, 18 expressed interest and agreed to participate in the study (75.00%). Of these 18, 15 completed at least part of the baseline survey (83.33%) and 12 completed both follow-up surveys (66.67%); note that only 14 were invited to complete follow-up surveys, contingent upon workshop attendance, which would be a proportion of 85.71% completion, 95%). Most participants attended at least one workshop (14; 77.78%), with 1 attending five sessions, 3 attending four, 4 attending three, and 5 attending one session (mean sessions attended = 2.5; SD = 1.45; median = 3). Demographic characteristics for the 15 participants who completed at least part of the baseline survey are in Table 1; participant flow is in Figure 1.

**Table 1:** Demographic characteristics.

Variable	Role	Mean (SD) or *N* (%)
Role	Physician/Primary Care Provider	6 (40.0%)
	Nurse Practitioner	2 (13.3%)
	Social Worker	2 (13.3%)
	Intensivist	2 (13.3%)
	Hospitalist	1 (6.7%)
	Physician/Primary Care Provider and ER	1 (6.7%)
Years in role		14.73 (11.10)
Race/Ethnicity	Non-Hispanic White	7 (46.7%)
	Non-Hispanic Black/African American	2 (13.3%)
	Non-Hispanic Asian	2 (13.3%)
	More than One	1 (6.7%)
	Hispanic/Latinx	2 (13.3%)

Demographic characteristics for the 15 participants who completed the baseline survey. One participant declined to report their professional role or race/ethnicity.

Of the 12 participants who responded to feasibility questions, most agreed that the intervention was appropriate for reducing bias in medicine (*n* = 8; 66.67%; Mean = 3.92, SD = 0.59), feasible (*n* = 10; 83.33%, Mean = 4.17, SD = 0.47), and acceptable (*n* = 9; 75.00%, Mean = 4.19, SD = 0.73). Results are also presented in Table 2, along with separate estimates for the 7 attending more than one session vs. the 5 attending one session only.

**Table 2:** Feasibility metrics.

Metric	Mean (SD) and *n* (%)	Overall (*n* = 12)	Attended more than one session (*n* = 7)	Attended one session only (*n* = 5)
Acceptable	Mean (SD)	4.19 (0.73)	4.07 (0.81)	4.35 (0.65)
	*n* (%)^a^	9 (75.0%)	5 (71.4%)	4 (80.0%)
Feasible	Mean (SD)	4.17 (0.47)	4.18 (0.35)	4.15 (0.65)
	*n* (%)^a^	10 (83.3%)	6 (85.7%)	4 (80.0%)
Appropriate	Mean (SD)	3.92 (0.59)	3.64 (0.48)	4.30 (0.54)
	*n* (%)^a^	8 (66.7%)	4 (57.1%)	4 (80.0%)

Feasibility metrics reported post-workshop among 12 participants who attended at least one narrative medicine workshop session.

^a^ Percent represents the number who agreed that narrative workshops were acceptable, feasible, or appropriate for reducing bias in medicine (mean score ≥4; possible range 1–5).

A total of 12 participants responded to the five open-ended questions and were included in the qualitative analysis. Two overarching themes were identified, with four subthemes each, corresponding to four of the five questions. The fifth question queried changes in ease in conversations about privilege and bias, and responses were coded as either agreeing or disagreeing. Codebook and definitions are in Table 3 (specific questions are above in the Measures section).

**Table 3:** Themes, subthemes, definitions, and illustrative examples.

Theme	Subtheme (question)	Definition
Communal compassion	Support and sharing^a^	Value of sharing in a group setting and receiving support from peers
	Connectedness and teambuilding^b^	Impact on ability to connect with peers on a personal and professional level
	Caring and community^c^	Caring and understanding, searching for common ground
	Finding one's voice^d^	Confidence, self-compassion, self-growth, and increased comfort with sharing one's own lived experience
Appreciation of diverse perspectives and lived experience	Hearing other's perspectives^a^	Impact of hearing others speak openly about their own perspectives and experiences
	Perspective taking and awareness of own biases^b^	Awareness of the power of one's own words to impact others, appreciation for other perspectives
	Appreciation of differences^c^	Appreciation for the different perspectives of peers with diverse lived experiences, appreciation of the chance to learn about others, which is not usually a part of the day-to-day work experience
	Listening and reflecting^d^	Importance of taking the time to listen to others and reflect
Increased ease in conversations	Agree^e^	
	Disagree^e^	

Reported by 12 participants who attended at least one narrative medicine workshop session.

^a^ Responses to the question: *Please tell me about a moment that stood out in one of the sessions of the narrative medicine workshop*.

^b^ Responses to the question: *What do you think changed, if anything as a result of having these sessions?*.

^c^ Responses to the question: *What did you learn about your team members in the process?*.

^d^ Responses to the question: *What did you learn about yourself in the process?*.

^e^ Responses to the question: *Do you think it is easier to have conversations of privilege and bias in your clinical assessment as a result of these sessions?*.

### Theme: communal compassion

Across all questions, workshop participants noted the unique group experience that, through sharing and communal support, helped build compassion, function more effectively as a team, and even find confidence in their own voice.

#### Support and sharing

Participants commented on the vulnerability they were able to achieve in the group setting. For example, participant 111 stated, “We were discussing a passage about being heard, and I shared a difficult time in my life when I was worried if I would be heard - a very personal story. I received a lot of support from other members of the workshop.” This deep level of openness and honesty was integral to creating a safe space to build community and support. As stated by participant 109, this was true even when topics were less personal, “The moment that stood out the most to me was in the first session attended where I immediately felt that I was among friends and we shared our reflections and writing on a beginning of a story that we all completed.”

#### Connectedness and teambuilding

Some participants stated explicitly that the workshops brought them closer together, as seen in comments by participant 108, “It brought faculty/participants closer together,” among others. Responses also clearly showed that this closeness had real-world consequences, with some being “more likely to ask my peers for guidance and collaboration” (participant 109), and others commenting on the importance of the workshops for creating connectedness and support within their team, “I think these sessions served allow us to continue take down barriers and in a safe space share personal stories that impact our lives. I think it reinforces work to support team members” (participant 111).

#### Caring and community

The support and connectedness fostered within the workshop highlighted not only diverse perspectives, but also the common and meaningful goals that brought them to the profession and to the workshop in the first place. Participant 102 wrote, “We have a lot in common. We all feel and care about what we do and we all want to do better.” This quote illustrates a search for common ground that is centered around caring and striving to do “better,” which opens the door to understanding and forgiveness during the tough moments in a high-stress environment. In other words, “it is important to remember our humanity in our busy day” (participant 112).

#### Finding one's voice

The support and connectedness within the workshop also enabled participants to gain confidence in ways they had not anticipated. This was noted by multiple participants, who found “That it is ok to write and share and to be open and let things flow” (participant 102) and learned “To be okay with vocalizing my thoughts which may be different from others” (participant 105). This suggests increased comfort with one's own unique perspective and an enablement of open and honest discussion without fear of repercussions.

### Theme: appreciation of diverse perspectives and lived experience

Intertwined with the ability to connect and support each other as a team was the common thread of understanding—particularly understanding differences in perspectives and personal histories. This appreciation of diversity allowed the supportive environment and teambuilding to take new depths, as participants empathized with each other, listened, and were heard.

#### Hearing other's perspectives

Participants found simply hearing different interpretations of the same piece of art or literature to be an eye-opening and impactful experience, even though this is relatively “low stakes” compared to sharing more personal histories. “Listening to others interpret the painting or the narrative. It made it clear how different people ‘see’ things while being exposed to the same situation or narrative” (participant 110). That said, some were clearly moved by their peers ability to open up and share personal histories, a practice that is relatively uncommon in the professional sphere. Participant 104 noted a moment that stood out to them, writing “When folks on the zoom session shared their own backgrounds - painful ones and personal details.”

#### Perspective taking and awareness of own biases

Hearing these differing perspectives led participants to have an increased awareness of how this shapes not only what a person says, but also the ways in which people hear and interpret them. Participant 110 captured this in their comment, stating, “I think I encourage myself to look at things from a broader perspective now before making a judgement or having an opinion.” A comment from participant 104 conveys increased awareness of the power of words to help or hurt: “More awareness of our words - on the [effect] they have on others, and to listen to not only the words, but the person these words are coming from.”

#### Appreciation of differences

Hearing others' stories and perspective taking helped participants to appreciate both personal and professional differences. They were able to see how these differences shaped people across personal and professional spheres, “Everyone in the group has many layers and great capacity to empathize but there is a burden that goes along with that from both our life stories and our roles with our patients and families” (participant 111). Participants pointed to the uniqueness of the opportunity to share and hear about these differences in the workshop. For example, participant 108 noted “That we all have rich lives and backgrounds, that are usually not shared in the professional workspace.”

#### Listening and reflecting

A key takeaway of the workshop for many participants was the simple realization that one should stop to listen and reflect before drawing conclusions. Participant 111 had a goal “To continue to slow down and listen to all of the voices around me […] Not to jump to a response.” This sentiment was shared by peers, as expressed by one participant who identified “That I need to listen more, be patient, and attuned” (participant 104).

### Theme: increased ease of conversations about privilege and bias

Of the 12 participants, 9 were coded as agreeing that the workshop sessions increased their ease in having conversations about privilege and bias in clinical assessment (75.0%). These agreements included additional important comments that illustrate the delicacy of these types of interactions and the need for all parties to be comfortable and prepared for such discussions. As stated by participant 109, “To some degree, yes. It will definitely facilitate these discussions with folks like the ones who attended these workshops and with whom I was able to connect around these readings. It has made me more receptive to uncertainty and the need to explore blind spots[,] but I do not think that all others are necessarily prepared for or interesting in those raw discussions.” Participant 108 highlighted the specific mechanisms by which these sessions facilitate difficult conversations, writing, “Yes. I think it encourages taking a step back and approaching an assessment from a trauma-informed perspective, with humility.” Of the three who disagreed, only participant 104 offered details regarding their answer, writing, “No. These conversations are never easy.”

## Discussion

Our study shows that narrative medicine workshops are feasible and show promise for reducing implicit bias in an interdisciplinary group of participants working with child abuse in a medical setting. Although our study cannot demonstrate in practice that narrative workshops reduce bias in reporting and diagnosing of child abuse, it provides a structure to mitigate bias, by creating a safe space to discuss difficult topic such as race, increasing awareness of one's own prejudice and stereotypes, viewing the perpective of others and allowing self-reflection. Furthermore it has the potential to reduce burnout which is a critical issue for clinicians working in the field.

Our findings provide support for the idea that close reading, writing to prompts, and group discussions result in awareness of one's own emotions, engagement with others, and recognition of the self and other as a potentially powerful strategy for tackling implicit bias in child abuse evaluations. Narrative medicine trains health care professionals to engage in close reading of texts and close listening to others, building capacity for attentive presence and non-judgmental reflection. The first step in clinician consultation for child abuse suspicion includes listening to a caretaker. Skills acquired in narrative medicine training will be critical to improve these interactions, for example, by paying attention to the details of the story provided, resisting the urgency to ask clarifying questions until the end of the interview, refraining from premature decision-making, and noticing what is left out of the story. Each of these steps is essential in establishing the plausibility and diagnosis of abuse (50).

Themes identified in our qualitative analysis showed a broad, positive impact of the narrative medicine workshops on interpersonal relationships in the workplace, including greater compassion and comfort in sharing their own experiences and a greater appreciation of diverse perspectives. These factors, in turn, contributed to a sense of community, support, and team cohesion that participants had not otherwise had the opportunity to experience. In addition to the skill of close reading, building team cohesion and appreciation is essential to reduce bias in child abuse evaluations. Asnes and colleagues note this team phenomenon as “harnessing the value of the multidisicplinary view” (50). Whether the team refers to the child protection team called in for consultation, the law enforcement-child protective services team investigating the case, the subspecialists consultants such as the radiologist or the neurosurgeon relied on to rule in or rule out alternate diagnoses, or the peer review team, team work is a key component of the quality of a child abuse consultation.

The deep sense of trust, safety and connectedness described by the workshop participants may also contribute to a decrease in burnout among professionals working in the child abuse field, as has been seen in other domains implementing narrative medicine strategies (e.g., oncology; 30). Unfortunately, high stress environments and schedules often do not allow the space for these types of trainings or discussion, yet these are the environments that need novel methods for building trust, safety, and connectedness the most. Although burnout was not identified directly in qualitative analyses, participants became more aware of their own biases without defensiveness, sharing in a common goal of caring and striving to do better in high stress. Other benefits of a narrative medicine approach include structural competency and empathy (28, 30).

For most participants, conversations on race and privilege were eased by the workshops, although it was recognized that these discussions are “never easy.” Of note, the child abuse service is an especially challenging setting for the frank expression and exposure of personal views regarding the clinical situations that must be discussed. Open conversation about parenting behaviors and safety of the home requires awareness of colleagues’ own loyalties and viewpoints, which engage deep and potentially divisive cultural identities. Child abuse consults to assess whether a family should be reported to child protective services are commonly triggered by specific parental behaviors (e.g., a parent who leaves an infant unattended on a bed, resulting in a fall and a skull fracture). Awareness of the ways in which the negative perceptions of the family may affect the intention of the person who initially calls to report the incident is critical and should be considered in abuse determinations.

Child abuse pediatricians may be asked in court about the specific steps they took to mitigate bias during their evaluation of child abuse (51). Optimally, bias and cognitive errrors such as premature closure (65) should be avoided (50). Our pilot data, indicating the acquisition of skills such as the ability to listen without interruption, to appreciate diverse perspectives, and to become aware of biases, as well as the cultivation team cohesion and trust, suggest that these goals may be achieved with narrative medicine. A systematic review by Milota et al. (35) found that narrative based interventions stimulate self-reflection and empathy and enable perspective taking, suggesting that narrative medicine-informed approaches may be particularly suited for easing conversations in these difficult clinical settings. The fact that effective implicit bias recognition and management (IBRM) trainings (52) share several characteristics with narrative medicine praxis provides further support for this proposition. IBRM trainings create a safe and nonjudgmental environment, flatten the hierarchy among participants, and normalize bias by reducing self-blame, building trust and enhancing comfort (53). Narrative medicine workshops create a safe and confidential space where stories illustrating inequality can be shared indirectly via work of art (e.g., text, poem, painting), which, like IBRM, avoids direct blame, allows self-reflection and taking in the perspectives of others, and decreases group hierarchy (36). Indeed, using art as a means of self-reflection facilitates self-distancing, which has shown to be helpful in anti-bias and antiracist trainings (42, 54).

Only 18 of 24 invited CAB-F members agreed to participate in the narrative workshops, and only 14 of these 18 attended at least one session—even after carefully selecting times for the workshop with the greatest participant availability. As such, there were clear challenges to recruitment and participation. Securing leadership commitment or providing Continuing Medical Education credits (CME) could encourage or incentive attendance. Allowing participants to attend during work hours, and providing these sessions for free, could also incentivize participation. Despite inability to commit to regular sessions and low attendance rates, participant ratings of feasibility metrics were highly positive. Compliance rates were high, as was agreement with the appropriateness, feasibility, and acceptability of implementing these workshops for reducing bias in medicine. Although none of the metrics were endorsed by 100%, both feasibility and acceptability met the generally accepted cut-off (75%) for continuing on to the next stage of intervention development (55), it is interesting that fewer people agreed that these workshops were appropriate for reducing bias among those who attended more than one session vs. more than one session only. However, it is impossible to draw conclusions from these data due to the small sample size. Future studies should test dose-response relationships and explore whether the appropriateness may become less obvious as the novelty of the material decreases. Qualitative comments demonstrate the participants' growth in listening to and appreciating the value of hearing personal responses to materials in the workshop.

These workshops are not enough in isolation, however, and one should be cautious in adopting narrative medicine training as the sole tool in the quest for social justice. Narrative medicine workshops will only address bias at the level of the individual provider, and they should be deployed alongside screening tools, clinical guidelines and pathways, and electronic health records (EHR) alerts to support clinical decision (56–58). Additionally, early Child Abuse Pediatrician engagement (when available; 12), and self-reflection (59) can enhance scope and efficacy of non-biased judgments in child abuse practice. Most importantly, without policy changes at the level of the institution, and without addressing structural racism in society at large, we run the risk of blaming marginalized communities for their behavior, putting the onus on the individual to solve the devastating and omnipresent problem of racial health disparities, and failing to effect lasting change (45).

## Limitations and future directions

There are several limitations to our study. The sample size was small, precluding our ability to detect significant changes in quantitative metrics (not reported). Generalizability across other settings and in more diverse samples is unclear, particularly given the small, self-selected sample, and because all sessions were conducted by a facilitator trained in narrative medicine. However, this sample size is in line with other pilot feasibility studies, and the goal was to inform the design of future studies that improve feasibility (e.g., by revising procedures to improve recruitment) and, eventually, studies that are powered to detect meaningful change in quantitative metrics at the level of the practitioner (e.g., bias, behaviors in a clinical setting), team (e.g., team cohesion), client (e.g., trust, clinical outcomes), and institution. Indeed, impacts at the hospital level could inform institutional and educational policies. Involving community stakeholders may result in a quicker path to action toward social justice. Other important endpoints that should be explored in future research include disparate evaluation and diagnosis of abuse and clinical outcomes in minoritized families, especially in locations and situations where suspicion of abusing a child can have dire outcomes of deportation or worse on immigrant families. These trials should additionally test hypothesized mechanisms of intervention effects (e.g., increased perspective taking). Future studies should recruit larger sample sizes across multiple institutions and include multiple facilitators in order to enhance external validity. They should include control groups, and they may also consider dose-response associations by exploring whether the impact of narrative medicine workshops is associated with the number of sessions attended. Despite the self-selected sample, it is important to note that, if narrative medicine workshops improve outcomes such as self-efficacy, reduced burnout, and bias reduction, there will still be a meaningful clinical impact on the patients treated by these practitioners—*even when practitioners self-select workshop participation*.

It should be noted that qualitative questions did not specifically elicit feedback on things that the participants did not like or that they would change about the workshops, which would have provided greater insight into feasibility and adapting methods for future implementation. It is possible that those participants who were more likely to be retained and respond to survey questions were also more likely to give a positive evaluation of the narrative medicine workshops, which could have biased responses.

This could have also contributed to the apparent discrepancy between positive evaluation of study feasibility and low attendance, which should be explored and addressed in future efforts to promote group cohesion and maximize exposure to workshop content. The challenges with respect to recruitment and session attendance may be mitigated by leadership actions discussed above (e.g., mandating attendance, providing CMEs). Because finding a common time for busy professionals to meet consistently can be challenging, collaboration with departmental leaders to schedule workshops into the workday may be a particularly effective solution. If efficacy is demonstrated in larger trials powered to detect significant effects of the intervention on quantitative metrics, these workshops could be incorporated as mandated anti-bias training.

Given that the narrative medicine workshops were designed collaboratively by faculty who themselves have had training in narrative medicine and sessions were facilitated by an experienced narrative medicine practitioner, adoption of the methods described in this report and their efficacy will hinge on the adequacy of training for those designing, implementing, and facilitating the intervention, suggesting a need for increasing training opportunities in this discipline. In addition, collaborating with the communities of patients in a settings' catchment area is critical for the integrity and success of this and other equity/justice interventions. Community-based organizations, committees of patient advocates, and community leaders will contribute to accuracy of understanding the positions of those served by the institution. Such collaborations can model the sharing of power often missing from institutional interventions. Conducting workshops with community members is another approach that has been successful in reducing stigma and prejudice (60). When community members participate alongside the providers responsible for making life-changing decisions, the potency of narrative medicine to mitigate disparities in child abuse may be even further enhanced.

## Conclusion

In conclusion, there is mounting evidence that the use of art and creativity allows for personal and structural insights on racism. Healthcare practitioners swear to “do no harm,” yet research shows that implicit biases can do just that. The field of medicine has an obligation to protect families. Narrative medicine workshops offer a promising tool to address implicit bias in medical settings. However, in order to decrease health disparities, implicit bias trainings will need to be coupled with strategies addressing structural racism (61). Fully powered trials that test the efficacy of implementing narrative medicine pedagogy in conjunction with other modes of anti-racist and anti-bias trainings are warranted.

## Data Availability

The raw data supporting the conclusions of this article will be made available by the authors, without undue reservation.
