# Supplementary material for: An exploratory study of addressing bias for child abuse teams: the role of narrative medicine
Source: Front Pediatr. 2026 Jan 14;13:1710240. doi: 10.3389/fped.2025.1710240 (PMC12847260; doi:10.3389/fped.2025.1710240)
Supplement: Supplementary file 1 [file Table1.docx]

**Appendix A**

| **Session** | **Creative Work** | **Writing Prompt** | **Themes** |
| --- | --- | --- | --- |
| 1 | Wade in the Water *Vocal performance by Sweet Honey in the Rock* | Write about your rivers. | Historical context of slavery to locate current racialized attitudes/actions; use of art form of music, gospel, and collective voices in harmony; exploration of body and voice expression; encouragement to see one’s own past in terms of justice and injustice. |
| 2 | won’t you celebrate with me  *Poem by Lucille Clifton* | Write about “shaping into a kind of life.” | Multiple traumas experienced by Clifton and resiliency theme. Theme of self-making vs culture/family-making. Encouragement to consider how one’s own “self” is made. |
| 3 | The Explorer  *Poem by Gwendolyn Brooks* | Write about the choices. | Brooks’s past history of advocacy; the poem elicits and contrasts multiple perspectives and interpretations of the words. Encourages focus on the mood of the poem, internal feelings, interiors, emotions, anxiety, and ambiguity. Connection to civil rights/school desegregation movements of the time. |
| 4 | Telegraph Avenue  *Novel by Michael Chabon* | “in a moment, maybe, the black boy would. . . . ” | Specific space of a segregated neighborhood; friendship between a black boy and a white boy; elements of embodiment and race from the start; possibilities of transracial cohesion; most fundamentally, possibility of seeing into and imagining a future. |
| 5 | Blood History  *Poem by Reginald Dwayne Betts* | Write about a time you were listened to. | Fractured families, absent black father, fatherhood. Black incarceration. Social cohesion among speaker and friends. Longing for stability and capacity to make one’s own meaningful future. |
| 6 | Emergency Room  *Painting by Robert Colescott* | Write about looking away *or* write about not looking away. | Painting of a hospital scene-very graphic surreal, racialized painting that displays repulsive bias assumptions that are never spoken but harbored by Whites about Blacks. |
